# QM/MM study of the binding of H_2_ to MoCu CO dehydrogenase: development and applications of improved H_2_ van der Waals parameters

**DOI:** 10.1007/s00894-020-04655-3

**Published:** 2021-02-04

**Authors:** Anna Rovaletti, Claudio Greco, Ulf Ryde

**Affiliations:** 1 Department of Earth and Environmental Sciences, Milano-Bicocca University, Piazza della Scienza 1, 20126 Milan, Italy; 2 Department of Theoretical Chemistry, Lund University, Chemical Centre, P.O. Box 124, SE-221 00 Lund, Sweden

**Keywords:** Hydrogenases, MoCu CO dehydrogenase, H_2_ oxidation, BigQM approach, QM/MM, Force field parametrization

## Abstract

The MoCu CO dehydrogenase enzyme not only transforms CO into CO_2_ but it can also oxidise H_2_. Even if its hydrogenase activity has been known for decades, a debate is ongoing on the most plausible mode for the binding of H_2_ to the enzyme active site and the hydrogen oxidation mechanism. In the present work, we provide a new perspective on the MoCu-CODH hydrogenase activity by improving the in silico description of the enzyme. Energy refinement—by means of the BigQM approach—was performed on the intermediates involved in the dihydrogen oxidation catalysis reported in our previously published work (Rovaletti, et al. “Theoretical Insights into the Aerobic Hydrogenase Activity of Molybdenum–Copper CO Dehydrogenase.” Inorganics 7 (2019) 135). A suboptimal description of the H_2_–HN(backbone) interaction was observed when the van der Waals parameters described in previous literature for H_2_ were employed. Therefore, a new set of van der Waals parameters is developed here in order to better describe the hydrogen–backbone interaction. They give rise to improved binding modes of H_2_ in the active site of MoCu CO dehydrogenase. Implications of the resulting outcomes for a better understanding of hydrogen oxidation catalysis mechanisms are proposed and discussed.

## Introduction

Hydrogenases represent a large enzyme family that catalyses the conversion of H_2_ to protons and electrons, as well as the reverse reaction [1]. They are all metalloenzymes and are subdivided into three main classes depending on the type of metal ions that compose their active site: [NiFe], [FeFe] and [Fe] hydrogenases. Still, also other metalloenzymes have the ability to oxidise H_2_ to protons and electrons, such as the MoCu CO dehydrogenase (MoCu-CODH, featuring a MoCu binuclear active site [2]) [3, 4]. In the latter case, the reducing equivalents of the oxidation reaction pass from the active site bimetallic centre via two [2Fe-2S] clusters to a FAD cofactor and they are finally transferred to the quinone pool of the electron transport chain [5, 6].

Many theoretical studies of hydrogenase reactivity have been published, based either on quantum mechanical (QM) cluster or on hybrid quantum mechanics/molecular mechanics (QM/MM) approaches [7–13]. Moreover, H_2_ and O_2_ gas diffusion inside the various protein matrices has been investigated via molecular dynamics (MD) simulations [14–17].

The accuracy of calculations at the molecular mechanics (MM) level critically depends on the accuracy of the employed force field (ff). Suboptimal ff parameters may give rise to incorrect conformations or misleading energies. This problem may also arise at the interface between the QM and MM subsystems in hybrid QM/MM calculations.

In the study of enzymes expressing hydrogenase activity, the relevance of employing accurate parameters for the H_2_–protein interaction is evident. In fact, gas diffusion towards or from the active site may correspond to a complex dynamic phenomenon that does not simply involve predefined channels but may be based on networks of dynamically and temporary formed pathways [18].

In a previous study, we proposed a mechanistic picture for the MoCu-CODH hydrogenase activity on the basis of QM/MM results [13]. As far as computed energies are concerned, it has been pointed out that both QM-cluster and QM/MM results may depend critically on the size of the QM system [19–25]. To tackle this issue, one of us has developed the BigQM approach within which stable energies are normally obtained using a large quantum model ($\sim $ 1000 atoms). The latter is composed by all groups within 4.5–6 Å from the QM system of the QM/MM calculation, all buried charged groups in the protein and by moving truncated groups three residues away from the active site [23]. A previous QM/MM study of the first steps of the CO oxidation reaction in MoCu-CODH [26] indicated that BigQM-based refinement of computed energies is a valuable approach also in the theoretical investigation of the latter enzyme.

In the present study, we have refined the QM/MM energies associated with the hydrogenase activity of MoCu-CODH. As described in the following, we found unexpected large differences in relative energies between the QM/MM and the BigQM results. We show that these large deviations are caused by poor van der Waals (VdW) parameters used for H_2_ which lead to much too short interactions with nearby backbone HN groups. Therefore, we have developed improved H_2_ VdW parameters, which correct the dubious interactions. A comparison with the results obtained with parameters previously described in literature shows that our developments represent a key step to obtain reliable energies and structures in the modelling of H_2_–protein interactions.

## Methods

### The MoCu-CODH protein

The setup of the protein was the same as in our previous QM/MM studies of MoCu-CODH [13]. All calculations were based on the crystal structure of CODH in its oxidised form (PDB ID: 1N5W) [2]. Only the active site-containing large subunit (L) of the MoCu-CODH dimer of LMS heterotrimers was considered. The setup of the protein involved a detailed analysis of all the protonable residues, based on calculations with PROPKA [27], studies of the hydrogen-bond pattern, the solvent accessibility and the possible formation of ionic pairs. Based on this, all Arg, Lys, Asp and Glu residues were considered in their charged form, with exception of Glu29 and Glu488 that were protonated on OE2 and Asp684 that was protonated on OD1. Cysteine ligands coordinating to metals were deprotonated. Histidine residues were assumed to be doubly protonated with exception of His177, 178, 210, 213, 243, 700, 753 and 788 that were protonated only on the NE2 atom and of His61, 339, 766 and 793 that were protonated on the ND1 atom. The protein was solvated with water molecules, forming a sphere with a 60-Å radius around the geometric centre. A 1-ns simulated-annealing molecular-dynamics simulation, followed by a minimisation, was run to optimise added protons and water molecules.

#### QM/MM calculations

The QM/MM calculations were performed using the ComQum software [28, 29]. The protein and the solvent were split into two subsystems: system 1 is the QM system (see Supporting Information) while system 2 contains the remaining part of the protein and the water molecules. During the QM/MM geometry minimisation, system 1 is described by a wavefunction and is relaxed by QM methods, whereas system 2 is represented by an array of partial point charges (electrostatic embedding) and is kept fixed at the crystallographic coordinates. Covalent bonds between the QM and MM systems were truncated using the hydrogen link-atom approach [30]. The QM system is capped with hydrogen atoms (hydrogen link atoms, HL), the position of which are linearly related to the corresponding carbon atoms (carbon link atoms, CL) in the full system. The latter are not included in the point-charge model. The total QM/MM energy in ComQum is calculated as [28, 29]

1 $$ E_{\text{QM/MM}} = E^{\text{HL}}_{\text{QM1+ptch2}} + E^{\text{CL}}_{\text{MM12},q_{1}=0} - E^{\text{HL}}_{\text{MM1},q_{1}=0}  $$

where E$^{\text {HL}}_{\text {QM1+ptch2}}$ is the QM energy of system 1 truncated by HL atoms and embedded in the set of point charges modelling system 2. E$^{\text {HL}}_{\text {MM1},q_{1}=0}$ is the MM energy of system 1, still truncated by HL atoms, without any electrostatic interactions. E$^{\text {CL}}_{\text {MM12},q_{1}=0}$ is the classical energy of the whole system, with CL atoms and with the charges in system 1 set to zero, in order to avoid double counting of the electrostatic interactions. Thus, ComQum employs a subtractive scheme with electrostatic embedding and van der Waals link-atom corrections [31].

The QM calculations were carried out at the BP86-D3(BJ)/def2-TZVP [32–35] level of theory using TURBOMOLE 7.2 software [36]. The resolution-of-identity technique was used to accelerate the calculations [37]. The MM calculations were carried out by means of the Amber software [38], using the Amber FF14SB force field for the protein [39] and the general Amber force field [40] with restrained electrostatic potential (RESP) charges [41] for the molybdopterin-cytosine dinucleotide (MCD) cofactor. Parameters used for H_2_ are discussed below. The two Fe_2_*S*_2_ clusters—fixed during the optimisation process—were described with RESP charges and a non-bonded model [42]. Single-point calculations at the B3LYP-D3(BJ)/def2-TZVPD [35, 43, 44] level were also run on the optimised geometries.

#### BigQM calculations

The BigQM technique was applied with the aim of obtaining more accurate QM/MM energies [23, 24] based on the knowledge that QM/MM calculations converge faster than QM-only ones, but models of about 1000 QM atoms are needed to obtain convergence of the energies [19, 21, 22, 25]. The minimal QM system (system 1) was extended with all chemical groups with at least one atom within 6.0 Å of it and junctions were moved three amino-acids away from each residue in the minimal QM system. In addition, all buried charges inside the protein were included, with exception of the two iron–sulfur clusters (see Supporting Information). The resulting BigQM model consisted of 983 (most calculations), 991 (when Cys388 is protonated) or 1019 atoms (when a water molecule is present in the active site). The BigQM calculations were performed on coordinates from the QM/MM optimisations, with a surrounding point-charge model, at the BP86-D3(BJ)/def2-SV(P) level. The multipole accelerated resolution-of-identity *J* approach (marij keyword) was employed to accelerate the calculations [45]. The resulting energies were corrected with a QM/MM MM term for the BigQM region:

2 $$ E_{\text{MM}} =E^{\text{CL}}_{\text{MM12},q_{1}=0} - E^{\text{HL}}_{\text{MM1},q_{1}=0}  $$

Finally, the energies were also corrected by taking into consideration the B3LYP-D3(BJ)/def2-TZVPD functional and basis set effects, using calculations with the standard QM/MM QM system with a point-charge model of the surroundings:

3 $$ E_{\text{corr}} =E^{\text{B3LYP/TZVPD}}_{\text{QM1,ptch2}} - E^{\text{BP86/SV(P)}}_{\text{QM1,ptch2}}  $$

### The H_2_–CH_3_*CONHCH*_3_ model

**Fig. 1:**
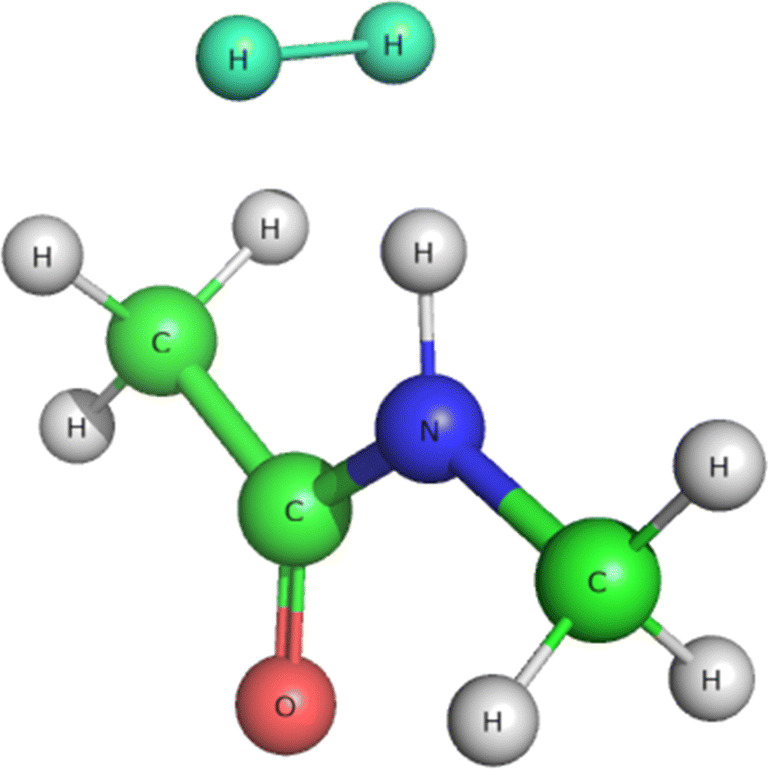
The small [H_2_ + CH_3_*CONHCH*_3_] model used for parametrization of the VdW parameters of H_2_. Colour code: blue, N; red, O; green, C; gray, H; cyan, H_2_ molecule

A small [H_2_ + CH_3_*CONHCH*_3_] model (see Fig. 1) was used to determine the potential-energy surface for the H_2_–HN interaction with QM methods and to subsequently determine van der Waals parameters to reproduce it.

The calculations were performed at the B3LYP-D3(BJ)/def2-TZVPD level using TURBOMOLE 7.2 software. The resolution-of-identity technique was employed to accelerate the calculations. The potential-energy surface for the H_2_–HN distance was scanned from a distance of 3.50 to 1.00 Å with steps of 0.25 Å, through optimisation of all degrees of freedom with exception of the analysed H–H distance that was kept fixed at the predetermined distance.

The Amber software was used to carry out all MM calculations in conjunction with Amber FF14SB force field for the CH_3_*CONHCH*_3_ protein backbone model. Restrained electrostatic potential (RESP) charges were obtained separately for H_2_ and CH_3_*CONHCH*_3_ running a single point energy calculation at HF/6-31G* [46–48] on the QM-optimised structure, followed by antechamber charge generation. Naturally, the charges on the two H atoms in H_2_ were zero. The H–H optimum bond length was set to 0.7443 Å with a force constant of 416.038 kcal/mol/Å^2^. Improved non-bonded parameters were obtained for the H_2_ molecule, as described in Section ?? ??–backbone non-bonded MM parameters.

## Results and discussion

### Evidence of inaccuracies in the QM/MM description of the H_2_–HN(backbone) interaction

In a previous study [13], we employed the hybrid QM/MM technique to investigate plausible mechanisms for the oxidation of H_2_ by MoCu-CODH. Binding of H_2_ to the active site was explored considering the enzyme resting state (**1H**), the presence of a water molecule in proximity to the copper ion (**2H**) and the hypothetical protonation of the Cu-bound cysteine residue (**3H**). Moreover, a variant of (**1H**) was considered in which Cu and the Mo–O_eq_ atom act as a frustrated Lewis pair (**1H-FLP**). For each of these variants, we studied possible H_2_ binding modes to the Cu centre, comparing the energy of the coordinated structures (hereafter indicated with an **R** tag) to the corresponding structure with the hydrogen molecule present in the active site but not linked to any atom (**NoB**). Deprotonation of the metal-activated H_2_ by means of the equatorial Mo-bound oxygen (referred to with **P** tag) or by an active-site glutamate residue (Glu763) (**P1**) was investigated. A second protonation of the equatorial Mo–O ligand was considered (**P2**) to form the fully reduced enzyme, Mo^IV^O(OH_2_)Cu^I^. Finally, the transition state for the proton-transfer reaction to the Mo-oxo ligand was located (**TS**) as well as the one involved in the transfer of the Cu-bound hydride to the Mo–OH_eq_ group (**TS2**).

With the aim of refining the QM/MM energies obtained for the intermediates, we performed BigQM calculations on the resulting optimised QM/MM geometries. Relative energies with respect to the **R** adducts are reported for the identified intermediates in Table 1.

**Table 1:** Relative energies (kcal/mol) calculated using the QM/MM and BigQM approaches at the B3LYP-D3(BJ)/def2-TZVPD level

		NoB	*R*	P1	TS	*P*	TS2	P2
QM/MM	1H	− 11.2	0.0	13.8		− 0.7	7.6	− 31.1
BigQM		− 38.3	0.0	0.2		− 42.0	− 32.3	− 64.2
QM/MM	2H	− 11.1	0.0	19.9				
BigQM		− 42.0	0.0	21.3				
QM/MM	3H	− 2.6	0.0		7.0	6.7	13.5	− 21.7
BigQM		15.0	0.0		4.5	3.6	12.4	− 10.9
QM/MM	1H-FLP	0.2	0.0		10.8	10.6	18.9	− 19.8
BigQM		10.0	0.0		7.0	6.3	16.0	− 15.9

Comparing the energy profiles obtained by the QM/MM and BigQM methods, large deviations are observed for the binding energies of H_2_ to Cu (${\varDelta }{\varDelta }\textit {E}_{\text {BigQM/QMMM}}^{\text {NoB/R}}$) for all of the tested active-site states (**1H**, **2H** and **3H**). The differences in relative energies were found to be 27, 31, 18 and 10 kcal/mol for **1H**, **2H**, **3H** and **1H-FLP**, respectively. Moreover, the **1H** species gave very large differences for all studied states, up 41 kcal/mol for the **1HR**/**1HP** pair. ${\varDelta }{\varDelta }\textit {E}_{\text {BigQM/QMMM}}^{\text {3HP2/3HR}}$ is also rather large (11 kcal/mol), whereas all other intermediates exhibit a difference in relative energy that is less than 5 kcal/mol, i.e. similar to what is observed for other systems [26].

To understand the origin of these large differences between the QM/MM and BigQM relative energies, we focused on the **1HR** → **1HP** step and calculated single-point BigQM energies at the BP86-D3(BJ)/def2-SV(P) level (i.e. without the additional corrections in Eq. 2) for QM systems of increasing size. Thus, the original BigQM system—composed of 983 atoms (see Section Methods)—was gradually reduced until the QM system of the QM/MM calculations was reached (46 atoms). The shrinking of the QM model was obtained by modulating three parameters for setting up the BigQM system (see Section Methods), i.e. the cutoff radius, the number of residues the junctions are moved away and whether charged buried residues are included. To reach the smallest models, the MCD cofactor was also truncated before or after the phosphate groups. The resulting energy differences between the **1HP** and **1HR** intermediates are reported in Table 2.

**Table 2:** Single point BigQM calculations with QM systems of different sizes

BigQM	*r*	*n*_aa_	ch aa	*n*_atoms_	Charge	*ΔE*
M	6	3	Yes	983	− 5	− 49.2
L	6	3	No	590	− 2	− 49.2
K	4	3	Yes	764	− 5	− 51.3
J	4	3	No	351	− 3	− 49.5
I	2	3	Yes	621	− 5	− 44.4
H	2	3	No	154	− 3	− 45.1
G	1	3	Yes	600	− 5	− 44.2
F	1	3	No	126	− 4	− 44.2
E	1	1	No	95	− 4	− 42.8
D	0	1	No	95	− 4	− 42.8
C	0	0	No	81	− 4	− 7.9
B	0	0	No	54	− 4	− 7.9
A	0	0	No	42	− 2	− 7.3
**QM/MM**	–	–	–	46	− 3	− 7.9

The models are obtained with varying cutoff radii (*r* in Å), varying number of residues the junctions are moved away (*n*_aa_) and whether buried charged residues are included (ch aa). The resulting QM systems are described by the total number of atoms (*n*_atoms_), the total charge (charge) and the relative energy of **1HP** with respect to **1HR** expressed in kcal/mol obtained at the BP86-D3(BJ)/def2-SV(P) level. The last line shows the same relative energy obtained in the standard QM/MM calculation. Models C–M include the entire MCD cofactor, whereas in B, MCD is modelled by the molybdopterin ligand plus the –PO_2_–O–PO_2_–OH group and in A, it is modelled by the molybdopterin ligand only, as in the original QM system of QM/MM calculations

**Fig. 2:**
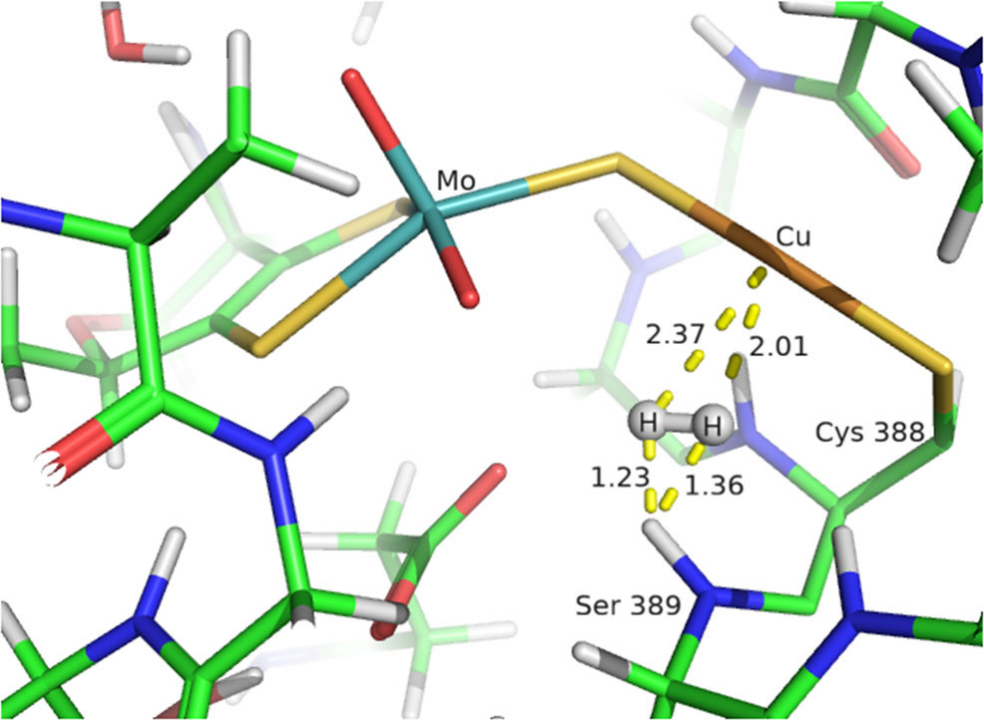
QM/MM optimised geometry of **1HR** focusing on the active site. Distances are in Å. Colour code: cyan, Mo; copper, Cu; yellow, S; blue, N; red, O; green, C; gray, H

It can be seen that BigQM energies change by less than 2 kcal/mol if the buried charged residues are included or excluded. Somewhat larger variations are observed for the various cutoff radii (43–51 kcal/mol), but the difference between the two largest radii is less than 2 kcal/mol. Truncating the MCD cofactor also has a minimal effect (less than 1 kcal/mol). Instead, the large difference comes from the number of residues the junctions are moved away. In the present case, this involves only the neighbours of the Cys388 residue. Reducing the number from 3 (in standard BigQM) to 1 has small effect (1.4 kcal/mol between models E and F). However, excluding also the last residue (from model D to C), *ΔE* suddenly jumps by 35 kcal/mol. By a careful analysis of intermediates **1HR** and **1HP** in the two models, we realised that the deviating energies arise from the very short H_2_–HN(Ser389) distances (1.23 and 1.36 Å) observed in the **1HR** geometry, as shown in Fig. 2.

**Fig. 3:**
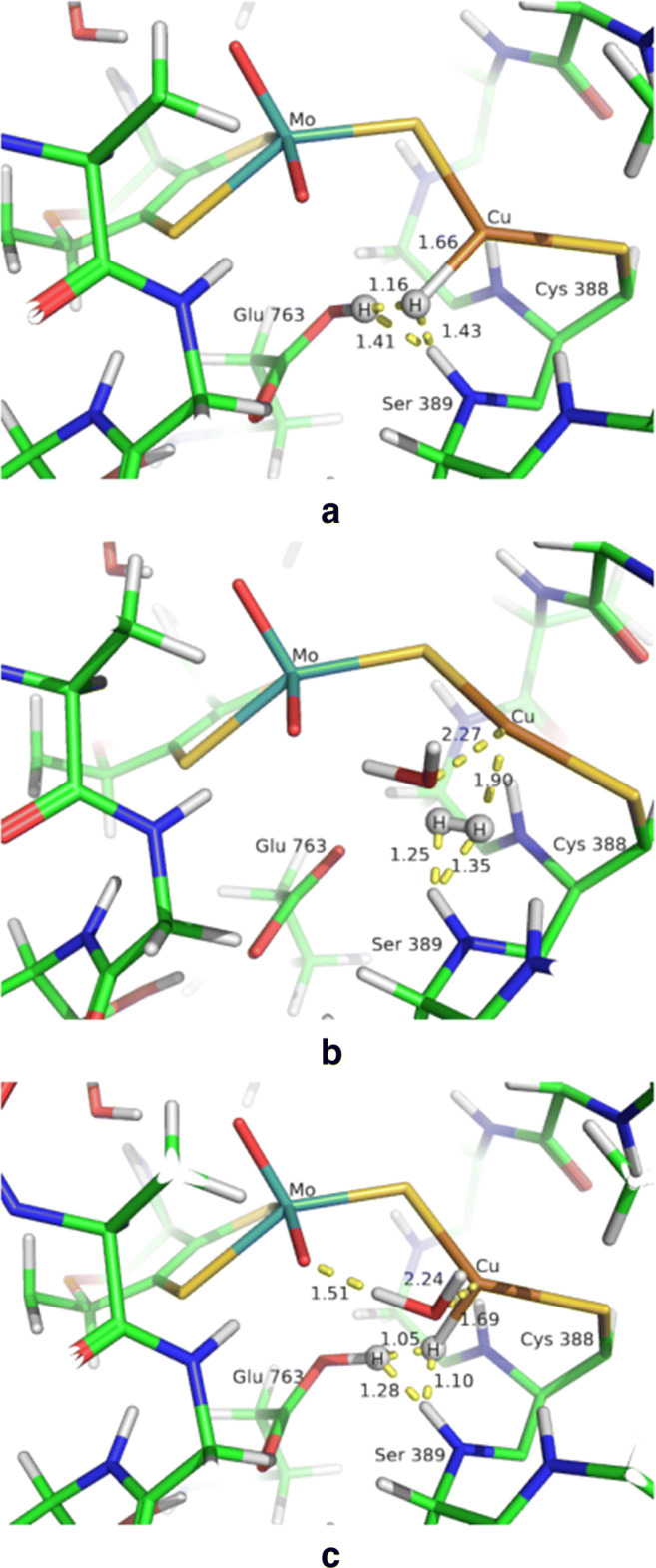
QM/MM optimised geometries for **(a)** **1HP1**, **(b)** **2HR** and **(c)** **2HP1**. All distances are in Å. The colour code is the same as Fig. 2

Based on this observation, we further analysed all the QM/MM optimised geometries and we found short H_2_–HN(backbone) interactions also for the **1HP1** (1.41, 1.43 Å), **2HR** (1.35, 1.25 Å) and **2HP1** (1.10, 1.28 Å) as shown in Fig. 3.

Such short H_2_–backbone distances indicates that the van der Waals repulsion between the peptide NH group and the hydrogen molecule is underestimated at the MM level (in these QM/MM calculations, H_2_ is in the QM system but Ser389 is in the MM system, so that their interactions are determined by the MM parameters, whereas in the BigQM calculation, both groups are in the QM system). This was examined in the following sections, by using a small [H_2_ + CH_3_*NHCOCH*_3_] model.

### Improving the H_2_–backbone non-bonded MM parameters

**Fig. 4:**
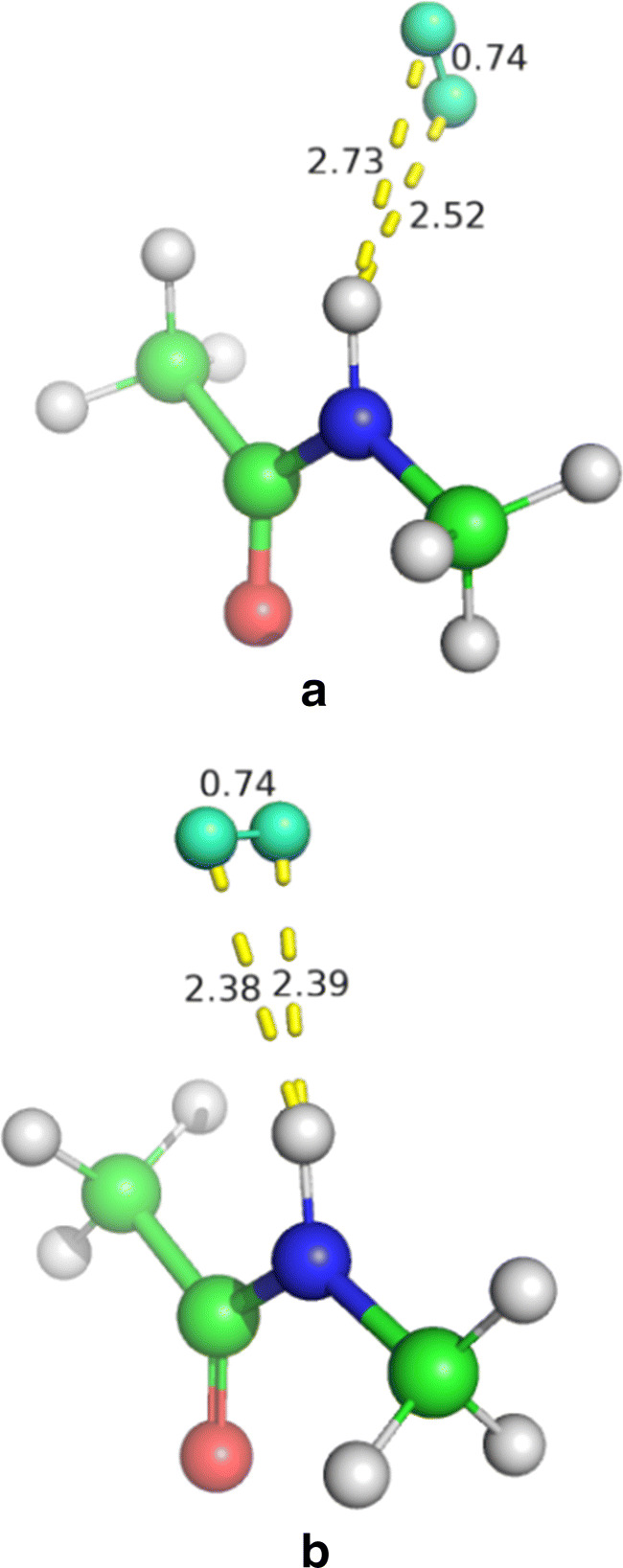
The [H_2_ + CH_3_*NHCOCH*_3_] model optimised at the B3LYP-D3(BJ)/def2-TZVPD level **a** and at MM level with the new VdW parameters **b**. All distances in Å. The colour code is the same as in Fig. 1

To confirm that the problem lies in the VdW parameters of H_2_, we optimised the structure of the [H_2_ + CH_3_*NHCOCH*_3_] model as described in Section Methods. The optimised geometry, reported in Fig. 4, shows H_2_–HN distances of 2.52 and 2.73 Å , i.e. much longer than the corresponding ones in the QM/MM structures of MoCu-CODH (see above). On the other hand, a full optimisation of the same [H_2_ + CH_3_*NHCOCH*_3_] model with the original MM parameters gave H_2_–HN distances of 1.49 and 1.50 Å . This shows that the original VdW parameters are suboptimal.

**Fig. 5:**
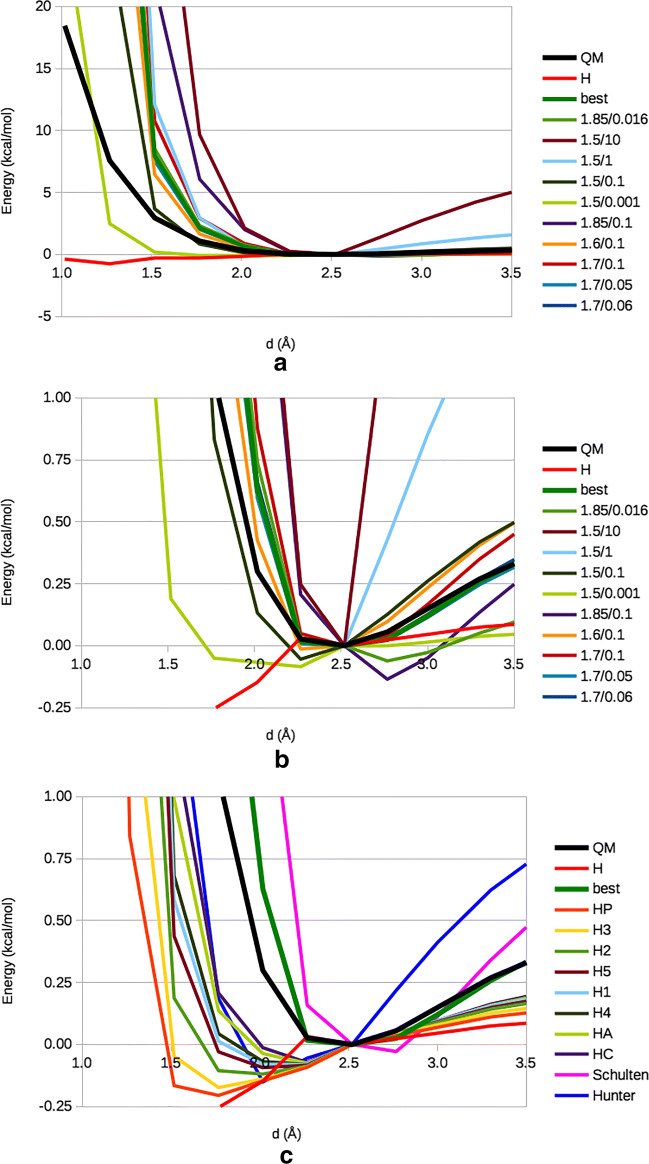
PES for the [H_2_ + CH_3_*NHCOCH*_3_] model obtained with QM and different MM force fields (those starting with H are the various AMBER atom types). The pairs of numbers in the legends indicate the *r*^∗^ and *𝜖* parameters in our calibration. “Schulten” and “Hunter” indicate the force fields suggested in refs. [15] and [49], respectively. “best” is our suggested force field

Starting from the QM structure, we performed a series of constrained optimisations in order to scan the potential-energy surface (PES) for the H_2_–HN interaction. The resulting QM PES is shown in Fig. 5a (black thick line).

The non-bonded interaction between two atoms at a distance of *r* is described in the Amber MM force-field as a sum of an attractive *r*^− 6^ (dispersion) and a repulsive *r*^− 12^ (exchange repulsion) term, according to the Lennard-Jones equation:

4 $$ E_{\text{VdW}}=\epsilon \left[ \left( \frac{r^{*}}{r} \right)^{12} - 2\left( \frac{r^{*}}{r} \right)^{6} \right] $$

where *r*^∗^ represents the distance at the energy minimum and *𝜖* defines the well depth.

The original VdW parameters for H in H_2_ were taken from the Amber FF14SB atom type H (H bound to N; the same parameters are used for H bound to S), with *r*^∗^ = 0.60 Å and *𝜖* = 0.0157 kcal/mol. They clearly give too short equilibrium distances and a not enough repulsive PES at short distances as is also shown in Fig. 5a (red line marked H; the MM PESs were calculated by single-point calculations to avoid any distortion of the structure or the influence from other MM parameters).

To obtain a better description of the PES, we tested different sets of VdW parameters (*r*^∗^= 1.50, 1.60, 1.70 and 1.85 Å, as well as *𝜖*= 0.01, 0.0157, 0.05, 0.055, 0.06, 1, 10 kcal/mol). The results are also included in Fig. 5a (lines marked with the values of *r*^∗^/*𝜖*) and they are described in Table 3. It can be seen that the optimum H(H_2_)–HN distance depends mainly on *r*^∗^, whereas *𝜖* is less important. It is also clear that it is not possible to reproduce both the long- and short-distance part of the PES (the MM potential is more repulsive than the QM one). We decided that it was most important to describe the minimum and longer distances, where the potential energy is close to the minimum energy and therefore often encountered in simulations. Figure 5b concentrates on that region. Employing the mean absolute deviation (MAD) in the interval *d* = 2.02—3.50 Å as the quality measure, we obtained optimum VdW parameters for *r*^∗^ = 1.7 Å and *𝜖* = 0.055 kcal/mol. With these, the MM curve reproduces the QM relative energies with a MAD of 0.06 kcal/mol (0.01 kcal/mol for distances 2.27–3.50 Å). At shorter distances, the curve is much too steep but this is acceptable because this part of the PES will rarely be visited in real simulations. A comparison of the optimum structure obtained with QM and the new MM parameters is shown in Fig. 4. It does not fully reproduce the distances observed in the QM structure because H_2_ moves to a more symmetric position in the MM structure and the methyl groups show different rotations.

**Table 3:** Performance of the various tested sets of H_2_ VdW parameters for the [H_2_ + CH_3_*NHCOCH*_3_] model

Set	*r*^∗^	*𝜖*	MAD	*Δd*	*ΔE*
1.85/0.016	1.85	0.0157	0.17	0.25	0.06
1.5/10	1.5	10	2.08	0.00	0.00
1.5/1	1.5	1	0.53	0.00	0.00
1.5/0.1	1.5	0.1	0.11	− 0.25	0.05
1.5/0.001	1.5	0.001	0.17	− 2.39	0.08
1.85/0.1	1.85	0.1	0.36	− 0.25	0.14
1.6/0.1	1.6	0.1	0.08	0.00	0.00
1.7/0.1	1.7	0.1	0.12	0.00	0.00
1.7/0.05	1.7	0.05	0.06	0.00	0.00
Best	1.7	0.055	0.06	0.00	0.00
1.7/0.06	1.7	0.06	0.06	0.00	0.00
H	0.6	0.0157	0.15	− 1.25	0.75
HP	1.1	0.0157	0.15	− 0.75	0.21
H3	1.187	0.0157	0.14	− 0.75	0.17
H2	1.287	0.0157	0.13	− 0.50	0.12
H5	1.359	0.015	0.12	− 0.50	0.09
H1	1.387	0.0157	0.12	− 0.25	0.08
H4	1.409	0.015	0.12	− 0.25	0.08
HA	1.459	0.015	0.11	− 0.25	0.08
HC	1.487	0.0157	0.10	− 0.25	0.07
Schulten	1.7682	0.1521	0.26	0.25	0.03
Hunter	1.5010	0.4769	0.24	− 0.50	0.15

The table lists the *r*^∗^ and *𝜖* parameters, as well as the mean absolute deviation (MAD in kcal/mol) of the resulting MM energy compared to the results obtained at the BP86-D3(BJ)/def2-TZVPD level in the range *d* = 2.02 − 3.50 Å, the error in the minimum distance (*Δd* in Å) and the error in the minimum energy (*ΔE* in kcal/mol)

The Amber FF14SB contains 13 sets of VdW parameters for different H atom types. Two of them (for water and OH groups) have zeroed parameters (the interaction is determined instead by VdW interactions by the heavy atom to which the H atom is bound), whereas the other have similar *𝜖* = 0.0157 or 0.0150 kcal/mol, but a varying *r*^∗^ = 0.6 − 1.487 Å (low values for H bound to polar atoms and high values for H bound to C). Our suggested parameters are both significantly larger than the AMBER VdW parameters. Figure 5c shows that all AMBER parameters give too short H_2_–HN distances (but the H atom type is by far the worst).

In MD simulations of the diffusion of H_2_ in hydrogenases [14–17], two different sets of VdW parameters have been used. One was suggested by Schulten and coworkers [15] and employs *r*^∗^ = 1.7682 Å and *𝜖* = 0.1521 kcal/mol. From Fig. 5c and Table 3, it can be seen that it gives a slightly too long H_2_–HN optimum distance and a too repulsive potential at longer distances. The other is a more sophisticated model by Hunter et al. [49]. In variance to all the other models, it employs an extra point at the H–H bond centre with a charge of –0.95 *e* and charges of 0.475 *e* on the H atoms (in order to give a proper quadrupole moment). Only the bond centre has non-zero VdW parameters, *r*^∗^ = 1.501 Å and *𝜖* = 0.4769 kcal/mol. In Fig. 5c and Table 3, it can be seen that it gives a too short H_2_–HN optimum distance and a too repulsive potential at longer distances.

Thus, we can conclude that the H_2_ interaction with the protein backbone NH group is sensitive to the VdW parameters and that only our suggested parameters give an accurate description of this interaction.

### Test case: H_2_ interaction with the MoCu-CODH active site

**Fig. 6:**
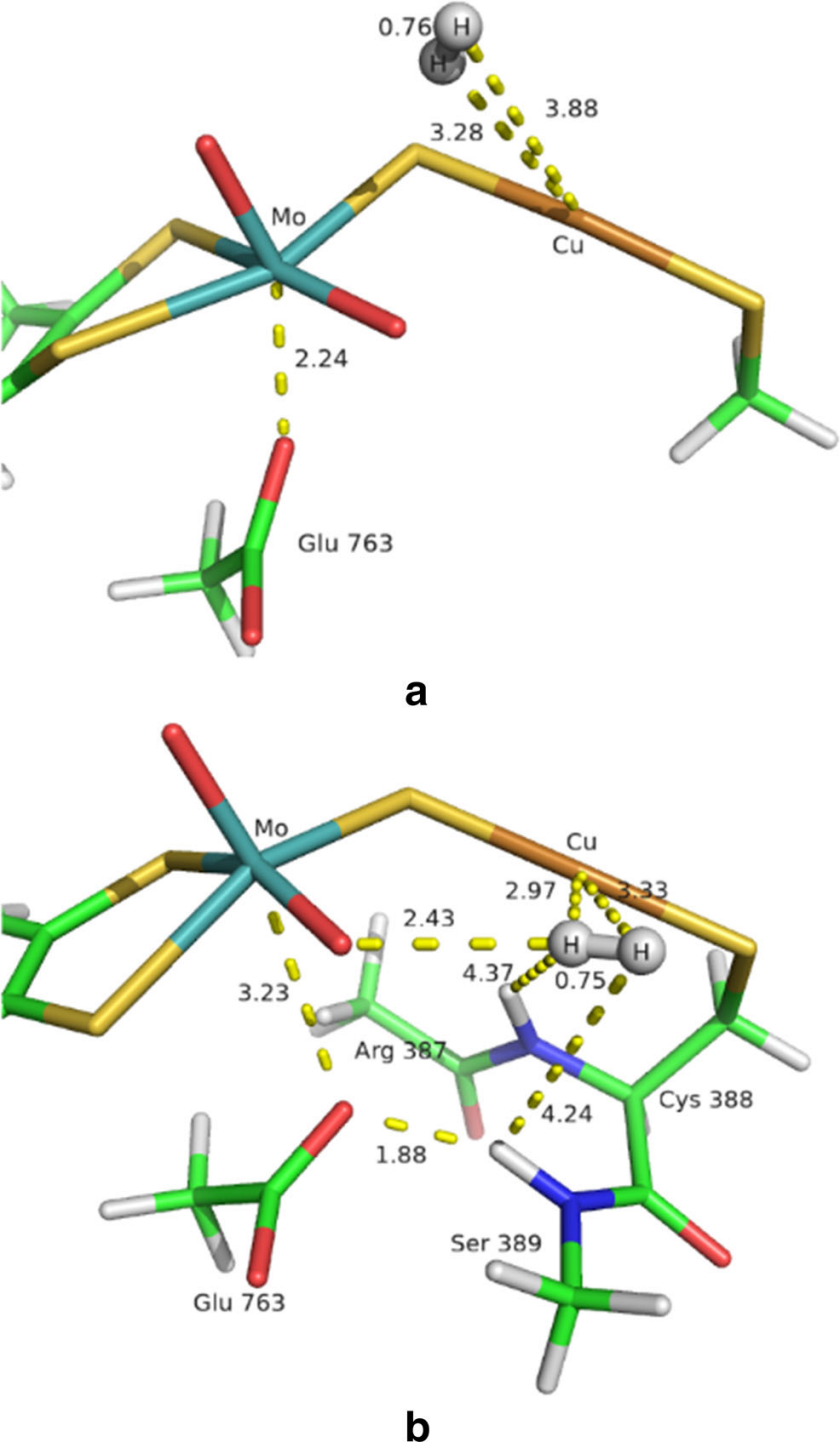
QM/MM optimised **1HR** at the BP86-D3(BJ)/def2-TZVP level obtained by employing the new VdW parameters, **1HR-A** **(a)**, or by including the backbone neighbouring Cys388 in the QM region of the QM/MM calculation, **1HR-B** **(b)**. All distances in Å. The colour code is the same as in Fig. 2

In order to validate the new set of VdW parameters, we run new QM/MM optimisations of the **1HR** intermediate. We tested two approaches. In the first (**1HR-A**), we used the QM system described in Section Methods but employed our new VdW parameters for H_2_ (*r*^∗^ = 1.7 Å and *𝜖* = 0.055 kcal/mol). In the second approach (**1HR-B**), the backbone neighbouring Cys388 was included in the QM system (see Figure S1), so that the H_2_–HN interaction was treated at a QM level. Both optimisations led to a complete detachment of H_2_ from the copper ion (see Fig. 6), with a Cu–H distance of 3.28, 3.88 Å and 2.97, 3.33 Å for **1HR-A** and **1HR-B**. The main difference between the two structures is that Glu763 coordinates to Mo in **1HR-A** with a Mo–O distance of 2.24 Å, whereas the Mo–O distance is 3.23 Å in the **1HR-B** structure, because it instead forms a hydrogen bond to the backbone HN group of Ser389, which is also introduced in the QM region. This leads to quite extensive changes in the local structure around the Mo ion. The new QM/MM optimisations show that the VdW parameters of H_2_ have a significant influence on the structure and that the **1HR** minimum previously obtained by means of the old VdW parameters for the H_2_ (Fig. 2) was an artefact caused by a bad description of the hydrogen–backbone interaction. Moreover, it shows that it is crucial to include the backbone around Cys388 in the QM system, with the two NH groups pointing into in the active site, providing potential hydrogen bonds to both Glu763 or various reaction intermediates. Apparently, the MM parameters are not accurate enough to model the subtle competition between Mo and the HN group of Ser389 for the carboxylate group of Glu763.

## Conclusions

In this paper, we have deepened our insights about the energetics of the putative initial steps for dihydrogen oxidation by MoCu CO dehydrogenase, keeping as a reference the QM/MM results from our previous investigation of the hydrogenase activity [13]. The energies were refined by using the BigQM approach on the QM/MM optimised geometries, analogously to our previous work on the CO-oxidation catalysis by this enzyme [26]. Unexpectedly, we observed large deviations in the relative energies obtained by the two approaches. Therefore, a detailed investigation was carried out to identify the cause of the deviation. We found that the reason was a suboptimal description of the H_2_–backbone interaction, caused by poor van der Waals parameters for H_2_. Using the simple H_2_ + CH_3_*NHCOCH*_3_ model, we show that none of the previously suggested VdW parameters for H_2_ is able to accurately reproduce the H_2_–HN(backbone) interaction, which has key relevance for a correct description of the binding of H_2_ in the active site pocket. Therefore, we have developed a new set of VdW parameters, with the aim of providing an accurate description of this interaction.

Based on the energy refinement work here presented, it is possible to exclude the previously proposed occurrence [4] of side-on H_2_ binding at the copper centre when the enzyme is in its resting state (**1HR**). Other routes for the initial interaction between substrate and enzyme active site need to be considered to account for the hydrogenase activity of Mo/Cu CO dehydrogenases, in line with previous theoretical investigations [13, 50].

From a methodological point of view, the BigQM approach is confirmed to be a highly valuable approach for the exploration of the complex potential energy surface associated with metalloenzymes activity. However, it is a single-point post-processing of the QM/MM energies. Therefore, it can identify problems in the QM/MM structures, but it cannot correct them. If the MM potential is poor, it needs to be improved or the QM system needs to be enlarged. Then, new QM/MM and BigQM calculations are needed to get reliable results. Naturally, accurate MM parameters are even more important for MD simulations.

Therefore, our new H_2_ parameters work will most likely be relevant also for future efforts towards the theoretical description of the interaction between dihydrogen and metalloenzymes for which H_2_ is a reagent, a product or both. The availability of improved sets of H_2_ interaction parameters may be relevant also for the role that computational chemistry will play in the context of the design of artificial hydrogenase enzymes, a challenging new frontier in biomimetics [51].

## References

[1] Lubitz W, Ogata H, Rüdiger O, Reijerse E (2014). Hydrogenases. Chemical reviews.

[2] Dobbek H, Gremer L, Kiefersauer R, Huber R, Meyer O (2002). Catalysis at a dinuclear [CuSMo(=O)OH] cluster in a CO dehydrogenase resolved at 1.1-Å resolution. Proceedings of the National Academy of Sciences.

[3] Santiago B, Meyer O (1996). Characterization of hydrogenase activities associated with the molybdenum CO dehydrogenase from Oligotropha carboxidovorans. FEMS microbiology letters.

[4] Wilcoxen J, Hille R (2013). The hydrogenase activity of the molybdenum/copper-containing carbon monoxide dehydrogenase of Oligotropha carboxidovorans. J Biol Chem.

[5] Wilcoxen J, Zhang B, Hille R (2011). Reaction of the molybdenum-and copper-containing carbon monoxide dehydrogenase from Oligotropha carboxydovorans with quinones. Biochemistry.

[6] Kalimuthu P, Petitgenet M, Niks D, Dingwall S, Harmer JR, Hille R, Bernhardt PV (2020). The oxidation-reduction and electrocatalytic properties of CO dehydrogenase from Oligotropha carboxidovorans. Biochimica et Biophysica Acta (BBA)-Bioenergetics.

[7] Siegbahn PEM, Tye JW, Hall MB (2007). Computational studies of [NiFe] and [FeFe] hydrogenases. Chem. Rev..

[8] Qui S, Xu Y, Shen S, Sun C (2020). Learning from nature: understanding hydrogenase enzyme using computational approach. WIREs Comput Mol Sci.

[9] Bertini L, Greco C, Bruschi M, Fantucci P, De Gioia L (2010). CO affinity and bonding properties of [FeFe] hydrogenase active site models. A DFT study. Organometallics.

[10] Breglia R, Greco C, Fantucci P, De Gioia L, Bruschi M (2018). Reactivation of the ready and unready oxidized states of [NiFe]-hydrogenases: mechanistic insights from DFT calculations. Inorganic chemistry.

[11] Greco C, Bruschi M, De Gioia L, Ryde U (2007). A QM/MM investigation of the activation and catalytic mechanism of Fe-only hydrogenases. Inorganic chemistry.

[12] Dong G, Phung QM, Pierloot K, Ryde U (2018). Reaction mechanism of [NiFe] hydrogenase studied by computational methods. Inorganic chemistry.

[13] Rovaletti A, Bruschi M, Moro G, Cosentino U, Greco C, Ryde U (2019). Theoretical insights into the aerobic hydrogenase Activity of Molybdenum–Copper CO Dehydrogenase. Inorganics.

[14] Cohen J, Kim K, King P, Seibert M, Schulten K (2005). Finding gas diffusion pathways in proteins: application to O2 and H2 transport in CpI [FeFe]-hydrogenase and the role of packing defects. Structure.

[15] Cohen J, Kim K, Posewitz M, Ghirardi ML, Schulten K, Seibert M, King P (2005). Molecular dynamics and experimental investigation of H2 and O2 diffusion in [Fe]-hydrogenase. Biochem Soc Trans.

[16] Teixeira VH, Baptista AM, Soares CM (2006). Pathways of H2 toward the active site of [NiFe]-hydrogenase. Biophysical journal.

[17] Topin J, Rousset M, Antonczak S, Golebiowski J (2012). Kinetics and thermodynamics of gas diffusion in a NiFe hydrogenase. Proteins: Structure, Function, and Bioinformatics.

[18] Wang P-H, Best RB, Blumberger J (2011). Multiscale simulation reveals multiple pathways for H2 and O2 transport in a [NiFe]-hydrogenase. J Am Chem Soc.

[19] Sumowski CV, Ochsenfeld C (2009). A convergence study of QM/MM isomerization energies with the selected size of the QM region for peptidic systems. The Journal of Physical Chemistry A.

[20] Hu LH, Eliasson J, Heimdal J, Ryde U (2009). Do quantum mechanical energies calculated for small models of protein-active sites converge?. J Phys Chem A.

[21] Liao R-Z, Thiel W (2013). Convergence in the QM-only and QM/MM modeling of enzymatic reactions: a case study for acetylene hydratase. Journal of computational chemistry.

[22] Flaig D, Beer M, Ochsenfeld C (2012). Convergence of electronic structure with the size of the QM region: example of QM/MM NMR shieldings. Journal of chemical theory and computation.

[23] Hu LH, Söderhjelm P, Ryde U (2012). Accurate reaction energies in proteins obtained by combining QM/MM and large QM calculations. J Chem Theory Comput.

[24] Sumner S, Söderhjelm P, Ryde U (2013). Effect of geometry optimizations on QM-cluster and QM/MM studies of reaction energies in proteins. J Chem Theory Comput.

[25] Roßbach S, Ochsenfeld C (2017). Influence of coupling and embedding schemes on QM size convergence in QM/MM approaches for the example of a proton transfer in DNA. Journal of chemical theory and computation.

[26] Rovaletti A, Bruschi M, Moro G, Cosentino U, Ryde U, Greco C (2019). A thiocarbonate sink on the enzymatic energy landscape of aerobic CO oxidation? Answers from DFT and QM/MM models of MoCu CO-dehydrogenases. J Catal.

[27] Olsson MHM, Søndergaard CR, Rostkowski M, Jensen JH (2011). PROPKA3: consistent treatment of internal and surface residues in empirical pKa predictions. Journal of chemical theory and computation.

[28] Ryde U (1996). The coordination of the catalytic zinc ion in alcohol dehydrogenase studied by combined quantum chemical and molecular mechanical calculations. J Comput Aid Mol Des.

[29] Ryde U, Olsson MH (2001). Structure, strain, and reorganization energy of blue copper models in the protein. Int J Quantum Chem.

[30] Reuter N, Dejaegere A, Maigret B, Karplus M (2000). Frontier bonds in QM/MM methods: a comparison of different approaches. The Journal of Physical Chemistry A.

[31] Cao L, Ryde U (2018). On the difference between additive and subtractive QM/MM calculations. Frontiers in chemistry.

[32] Becke AD (1988). Density-functional exchange-energy approximation with correct asymptotic behavior. Physical review A.

[33] Perdew JP (1986). Density-functional approximation for the correlation energy of the inhomogeneous electron gas. Phys Rev B.

[34] Grimme S, Ehrlich S, Goerigk L (2011). Effect of the damping function in dispersion corrected density functional theory. Journal of computational chemistry.

[35] Weigend F, Ahlrichs R (2005). Balanced basis sets of split valence, triple zeta valence and quadruple zeta valence quality for H to Rn: Design and assessment of accuracy. Phys Chem Chem Phys.

[36] TURBOMOLE V7-2 (2017) A development of University of Karlsruhe and Forschungszentrum Karlsruhe GmbH, 1989–2007, TURBOMOLE GmbH, since 2007. http://www.turbomole.com

[37] Eichkorn K, Weigend F, Treutler O, Ahlrichs R (1997). Auxiliary basis sets for main row atoms and transition metals and their use to approximate Coulomb potentials. Theoretical Chemistry Accounts: Theory, Computation, and Modeling (Theoretica Chimica Acta).

[38] Case DA, Babin V, Berryman J, Betz RM, Cai Q, Cerutti DS, Cheatham Iii TE, Darden TA, Duke RE, Gohlke H, et al. (2014) Amber 14

[39] Maier JA, Martinez C, Kasavajhala K, Wickstrom L, Hauser KE, Simmerling C (2015). ff14SB: improving the accuracy of protein side chain and backbone parameters from ff99SB. Journal of chemical theory and computation.

[40] Wang J, Wolf RM, Caldwell JW, Kollman PA, A. CD (2004). Development and testing of a general Amber force field. J Comput Chem.

[41] Bayly CI, Cieplak P, Cornell WD, Kollman PA (1993). A well-behaved electrostatic potential based method using charge restraints for deriving atomic charges: the RESP model. J Phys Chem.

[42] Hu L, Ryde U (2011). Comparison of methods to obtain force-field parameters for metal sites. J. Chem. Theory Comput..

[43] Becke AD (1993). A new mixing of Hartree–Fock and local density-functional theories. The Journal of chemical physics.

[44] Lee C, Yang W, Parr RG (1988). Development of the Colle-Salvetti correlation-energy formula into a functional of the electron density. Physical review B.

[45] Sierka M, Hogekamp A, Ahlrichs R (2003). Fast evaluation of the Coulomb potential for electron densities using multipole accelerated resolution of identity approximation. The Journal of chemical physics.

[46] Hartree DR (1928) Proc. cambridge soc. vol 24, pp 89–111

[47] Fock V (1930). Näherungsmethode zur lösung des quantenmechanischen mehrkörperproblems. Zeitschrift für Physik.

[48] Hehre WJ, Radom L, Pople JA, Schleyer PR (1986) Ab initio molecular orbital theory

[49] Hunter JE, Taylor DG, Strauss HL (1992). Calculation of the rotational Raman spectrum of H2 dissolved in water. J Chem Phys.

[50] Breglia R, Bruschi M, Cosentino U, De Gioia L, Greco C, Miyake T, Moro G (2017). A theoretical study on the reactivity of the Mo/Cu-containing carbon monoxide dehydrogenase with dihydrogen. Protein Engineering, Design and Selection.

[51] Simmons TR, Berggren G, Bacchi M, Fontecave M, Artero V (2014). Mimicking hydrogenases: from biomimetics to artificial enzymes. Coordination chemistry reviews.

